# EEG analysis of the visual motion activated vection network in left- and right-handers

**DOI:** 10.1038/s41598-022-21824-x

**Published:** 2022-11-15

**Authors:** Michaela McAssey, Thomas Brandt, Marianne Dieterich

**Affiliations:** 1 Department of Neurology, University Hospital, Ludwig-Maximilians-Universität, München, Germany; 2 German Center for Vertigo and Balance Disorders-IFB, University Hospital, Ludwig-Maximilians-Universität, München, Germany; 3 Graduate School of Systemic Neurosciences (GSN), Ludwig-Maximilians-Universität, München, Germany; 4 RTG 2175, Perception in Context and its Neural Basis, Ludwig-Maximilians-Universität, München, Germany; 5 Munich Cluster for Systems Neurology (SyNergy), München, Germany

**Keywords:** Human behaviour, Perception, Sensory processing

## Abstract

Visually-induced self-motion perception (vection) relies on interaction of the visual and vestibular systems. Neuroimaging studies have identified a lateralization of the thalamo-cortical multisensory vestibular network, with left-handers exhibiting a dominance of the left hemisphere and right-handers exhibiting a dominance of the right hemisphere. Using electroencephalography (EEG), we compare the early processing of a vection-consistent visual motion stimulus against a vection-inconsistent stimulus, to investigate the temporal activation of the vection network by visual motion stimulation and the lateralization of these processes in left- versus right-handers. In both groups, vection-consistent stimulation evoked attenuated central event-related potentials (ERPs) in an early (160–220 ms) and a late (260–300 ms) time window. Differences in estimated source activity were found across visual, sensorimotor, and multisensory vestibular cortex in the early window, and were observed primarily in the posterior cingulate, retrosplenial cortex, and precuneus in the late window. Group comparisons revealed a larger ERP condition difference (i.e. vection-consistent stimulation minus vection-inconsistent stimulation) in left-handers, which was accompanied by group differences in the cingulate sulcus visual (CSv) area. Together, these results suggest that handedness may influence ERP responses and activity in area CSv during vection-consistent and vection-inconsistent visual motion stimulation.

## Introduction

Perception of self-motion relies on the integration of information from multiple modalities including the visual, vestibular, somatosensory, and auditory sensory systems. Although each system relays information relevant for determining self-motion perception, the visual system exerts a dominant influence. This is demonstrated by the fact that a physically stationary observer experiences an erroneous sensation of apparent self-motion, called visually induced vection, when exposed to large-field visual motion stimulation^1^. Vection typically takes several seconds to develop following motion onset and perception tends to fluctuate between periods of vection and object-motion perception during prolonged motion exposure^1^. Further, the characteristics of vection perception are largely subjective, with the same visual motion stimulus often generating highly variable onset latencies and strength/intensity reports both between and within individuals^2,3^.

Several positron emission tomography (PET) and functional magnetic resonance imaging (fMRI) studies have identified a large cortical network involved in vection perception. Early imaging studies reported an activation of primary and secondary visual cortices along with concurrent deactivation of multisensory vestibular cortex—primarily in the parieto-insular vestibular cortex (PIVC)—during exposure to vection-inducing motion stimulation^4–6^. This activation-deactivation pattern is thought to reflect an inhibitory visual-vestibular interaction mechanism for self-motion perception, which enables the dominant sensorial weight to shift from one sensory modality to another, presently more reliable modality^4,7^. While this activation-deactivation pattern is associated with vection perception, it is not solely responsible for, nor indicative of vection. Indeed, several studies have observed similar activation-deactivation patterns in visual and vestibular networks, regardless of vection presence^8–10^. Such observations indicate that the PIVC likely responds to motion stimulation in general, with deactivations encoding an absence of primary vestibular input during vection-compatible visual motion stimulation^9,11^. This information from the PIVC feeds into the wider multisensory cortical vestibular network, contributing to the visual-vestibular interaction underlying vection.

In line with the hypothesis that vection perception involves visual-vestibular interaction, vection-specific activity has been located in the superior parietal lobule/precuneus, the anterior cingulate gyrus, the right post-central region and the cerebellar vermis^9^. Further, a recent review of the literature identified a probable cortical vection network, including visual area V3, motion area V6, area MT+ and the superior middle temporal area (MST), the ventral intra-parietal area (VIP), the parieto-insular vestibular cortex (PIVC), and the visual area of the cingulate sulcus (CSv)^12^. This cortical vection network overlaps substantially with a proposed network for cortical optic flow parsing^13^. This latter network identifies the CSv area, cingulate motor area (CMA), parieto-insular cortex (PIC), and lateral occipital region (LOR) as regions that not only prefer self-motion information, but also respond negatively to object-motion information. Further, this network identifies an important role of the precuneus and cingulate region in the processing of ego-motion information^13^.

While early PET/fMRI studies of vection have contributed much to our understanding of vection, the resulting findings are constrained by several methodological limitations. For example, the relatively low temporal resolution offered by these methods may fail to capture core aspects of vection, such as the brain processes involved in the relatively rapid alternations between object- and self-motion perception. Further, such studies often require participants to lie supine, which itself alters the visual-vestibular conflict with respect to the gravity vector (e.g., a sensory mismatch between visually induced continuous apparent self-rotation in yaw and a limited body tilt by graviceptive otolith input about true verticality). Lastly, the use of MRI itself may activate the vestibular system^14,15^, thus extra care must be taken to ensure that effects observed in the multisensory cortical vestibular network can be attributed to vection specifically.

Electroencephalography (EEG) not only circumvents these methodological limitations but may also prove useful for identifying objective neural markers of vection^16,17^. For example, initial EEG studies have identified a role of alpha band oscillatory activity in vection perception^18–22^, with some evidence to suggest that alpha activity decreases around the time of vection onset^20,21^. Further, an association has also been reported between the intensity of vection perception and oscillatory activity in the delta, alpha, and beta bands^22^. Studies investigating early event-related potential (ERP) responses to visual motion stimuli that subsequently induce vection under prolonged exposure have indicated a potential relationship between N2 component amplitude and the subjective experience of vection^23–25^.

While traditional neuroimaging and EEG approaches have both yielded insight into the neural mechanisms of vection perception, attempts to reconcile and integrate findings from both approaches are lacking. One potential explanation for this is that  localizing the source of EEG activity obtained at the scalp is a challenging and ill-posed problem. However, several algorithms to estimate the location of EEG sources now exist. One such algorithm is the low-resolution brain electromagnetic tomography algorithm (LORETA), whose accuracy has been demonstrated by EEG-PET^26^ and EEG-fMRI^27^ studies, including in spatially complex regions like the insula^28^, which forms a core part of the multisensory cortical vestibular network.

The present study combines ERPs and source localization (eLORETA) to investigate the early neural processing of a coherent motion stimulus that induces vection under prolonged exposure, against that of a vection-inconsistent control stimulus. Furthermore, although a lateralization of the thalamo-cortical multisensory network is well established, with left-handers exhibiting left hemisphere dominance and right-handers exhibiting right hemisphere dominance^29–35^, it is not yet clear if the visual-vestibular interaction underlying vection perception is affected by the lateralization of multisensory vestibular processing. The few studies investigating handedness-dependent modulations of vection perception and/or processing have yielded mixed evidence, with one study reporting differences in vection perception between left- and right-handers^36^ and another reporting differences in EEG measures of vection-related processing but comparable vection perception between the two groups^21^. Consequently, the present study has two major aims: (1) to disclose the temporal activation of the vection network by visual motion stimulation, and (2) to examine the potential hemispheric lateralization of these processes in left- versus right-handers.

## Methods

### Participants

Thirty-five right-handed (17 female, mean age: 27.69 years, SD: 3.94) and thirty left-handed (22 female, mean age: 25.27 years, SD: 3.96) healthy adults participated in the study. All participants reported normal or corrected-to-normal vision and no prior history of vestibular or neurological disorders. The 10-item Edinburgh Handedness Inventory^37^ was used to determine handedness for each participant (right handers: 5.7% ≥  + 40, 11.4% ≥  + 60, 8.6% ≥  + 70, 17.1% ≥  + 80, 57.1% ≥  + 90; left handers: 6.6% ≥ -40, 3.3% ≥ -50, 23.3% ≥ -60, 23.3% ≥ -70, 20% ≥ -80, 23.3% ≥ -90). Experimental protocols were approved by the local ethics committee at the ittee at the Ludwig-Maximilians-Universität, München, Germany and all methods were carried out in accordance with the Declaration of Helsinki. All participants gave their informed written consent prior to their participation and received either financial compensation (€10/hour) or partial course credit.

Following initial preprocessing, 6 right-handed and 2 left-handed participants were excluded from the EEG analyses due to poor data quality (i.e. fewer than 30 artefact-free trials per condition). This resulted in a final EEG sample size of 29 right-handed and 28 left-handed participants. No participants were excluded from the behavioural analyses. Part of the data from the first 25 right-handers and 25 left-handers was included in separate analyses as part of a different study^21^.

### Visual motion stimulation

The visual motion stimulation used in the present experiment is identical to that reported in our previous study^21^. In brief, the stimuli comprised two movies: a coherent and an incoherent pattern of rotating dots. Both stimuli consisted of 1000 randomly spaced white dots on a black background, with a central green dot as the fixation point. The dots rotated in the roll plane in either a clockwise (CW) or counter-clockwise (CCW) direction, at a velocity of 30°/s. In the coherent condition, the rotation of the dots followed a smooth, circular trajectory. In the incoherent condition, each dot had a random sinusoidal movement in both the X and Y direction added to the overall circular trajectory (i.e. the phase and amplitude of the additional sinusoidal movement was randomized separately for each dot). Consequently, each individual dot appeared to move in a random path, despite the global pattern maintaining a (CW or CCW) circular trajectory and a mean global velocity of 30°/s. The stimuli were created in MATLAB (The MathWorks Inc., Natick, MA, USA) using Psychophysics Toolbox extensions^38–40^ and were projected onto a custom-built dome with a diameter of 75 cm (see Supplementary Fig. S1 for schematic illustration of experimental setup). The distance between the apex of the dome and the participant’s nasion was 31 cm. The stimuli rotated around the line of sight, subtending a visual angle of 100°. The experiment was conducted in a dark room.

### Experimental procedure

For each participant the experimental apparatus was adjusted to ensure that the dome-centre and line of sight were aligned. On each trial, the presented dots first appeared stationary for a jittered period (3–5 s), before rotating (20 s) and then returning to stationary (10 s). Participants reported perceived vection onset and offset by making button presses on a gaming controller. Separate buttons denoted perceived CW and perceived CCW vection onset/offset. Participants held the controller in both hands and used both middle and index fingers to make responses. At the end of each trial participants were asked to verbally rate the strength of their vection experience on a scale of 0–10, where 0 is ‘no vection’ and 10 is ‘I felt I was really moving’. The response was recorded by the experimenter. Participants were seated with their head on a chin rest and were instructed to maintain fixation on the central green dot for the duration of each trial. Participants were also instructed to avoid following the moving dots with their eyes. Each participant completed 100 trials: 50 coherent trials and 50 incoherent trials, each with 25 trials in CW and CCW directions. Trial order was random, with trials presented in blocks of 10. Participants were encouraged to take a self-timed break at the end of each block, and between trials if necessary, in order to prevent fatigue.

Prior to the main experiment, each participant completed a short practice block comprising 12 trials presented in a random order (6 trials per condition, with 3 in each direction). The practice allowed participants to become familiar with the experimental task and to self-calibrate their use of the vection strength scale.

### EEG acquisition

The EEG was recorded using a 64 active electrode system (EASYCAP and BrainProducts, GmbH, Germany) with BrainVision Recorder software (BrainProducts, GmbH, Germany). Electrodes were fixed to standardized elastic caps following the international 10–10 system. The reference electrode was positioned at FCz. Bipolar electrode montages were used to record horizontal and vertical eye movements (i.e. EOG). Data were collected at a sampling rate of 1000 Hz, with no additional online filters. Impedances were kept below 5 kΩ throughout the recording. The EEG, visual motion stimulation, and response controller were synchronized using triggers sent via parallel port to the EEG recording. This enabled the accurate calculation of vection onset latency and duration based on participant button presses, and the computation of ERPs that were time-locked to stimulus motion onset.

### EEG preprocessing and ERP computation

Preprocessing and analysis were performed using the EEGLAB toolbox^41^ and custom MATLAB scripts. A 50 Hz and 100 Hz notch filter was applied to remove line noise using the CleanLine plugin^42^. A 0.1 Hz high-pass filter and a 30 Hz low-pass filter were then applied before re-referencing the data to the common average. The data were segmented into 600 ms epochs, ranging from − 200 ms to + 400 ms, relative to stimulus motion onset. The 200 ms pre-stimulus interval served as the baseline period for baseline correction. Epochs containing eye blinks, eye movements, or excessive signal range were excluded from analysis. To identify epochs for rejection, each epoch was segmented into a 200 ms window using a sliding window moving at 50 ms intervals. An epoch was rejected if any given 200 ms window was found to contain (a) a standard deviation greater than 35 µV in the EOG, Fp1, Fp2, or Fz electrodes or (b) if the signal range exceeded 100 µV. To ensure a sufficiently high signal-to-noise ratio of the ERP averages, trials in the CW and CCW directions were collapsed within the coherent and incoherent conditions. Participants were excluded from EEG analyses if fewer than 30 artefact-free epochs (i.e. trials), in two or more electrodes, were obtained for either the coherent or the incoherent condition. This resulted in the exclusion of 6 right-handed and 2 left-handed participants from the EEG analyses.

Grand average ERPs, time-locked to stimulus motion onset, were computed separately for the coherent and incoherent conditions, for left- and right-handers respectively. Condition difference waves (i.e. coherent minus incoherent) were also computed separately for both groups. Following visual inspection, two time windows were identified for further analysis: an early window ranging from 160 to 220 ms and a later window ranging from 260 to 300 ms. Mean amplitude within these time windows was calculated at each electrode, for coherent and incoherent conditions, as well as for difference waves, for both left- and right-handers.

### Source localization (eLORETA)

The exact low-resolution brain electromagnetic tomography algorithm (eLORETA) developed and implemented by Pascual-Marqui^43,44^, and freely available from the LORETA webpage (http://www.uzh.ch/keyinst/loreta.htm), was used to estimate the most likely generators of the observed ERP signals. For both conditions, the average ERP time series of each participant were exported to LORETA and a transformation matrix was applied. The first analysis aimed to identify generator differences between the coherent and incoherent conditions, for left- and right-handers respectively. To this end, the mean activity in the early and late windows was compared between conditions, separately for left- and right-handers. Significant effects were tested using paired-group t-statistic contrasts (5000 randomisations SnPM, significance threshold of *p* < 0.05). A second analysis aimed to identify generators that might explain differences between left- and right-hander condition difference waves. An independent groups test was conducted, (t-statistic, 5000 randomisations SnPM, significance threshold of *p* < 0.05), comparing the coherent minus incoherent localised activity of right-handers to that of left-handers. Again, tests were conducted for the mean over early and late windows. Suprathreshold voxels in each analysis were labelled in MATLAB using the mni2atlas tool^45^ and the Juelich histological and Harvard–Oxford atlases as implemented in FSL^46^. Our results were compared against those in previous studies for area CSv^47^ and the retrosplenial cortex^48,49^.

### Statistical analyses

Statistical analyses were conducted in MATLAB. Figures were created using custom MATLAB scripts, with the cbrewer tool^50^, and MRIcroGL^51^.

#### Behavioural data

The following behavioural data were obtained on each trial: (1) vection presence, i.e. if vection was reported, (2) onset latency, i.e. the time between motion onset and vection onset, (3) duration, i.e. how long a period of vection lasted, and (4) vection strength, i.e. subjective rating of how strong the vection experience was. In-line with the EEG analyses, CW and CCW trials were collapsed within coherent and incoherent conditions. For each behavioural measure, potential differences between left- and right-handers were assessed using the non-parametric Wilcoxon rank sum test. Separate tests were conducted for coherent and incoherent conditions, using Bonferroni corrections to address the problem of multiple comparisons. Effect sizes were calculated as Z/√(number of observations).

#### ERPs

Cluster-based permutation tests^52,53^ were conducted to test for effects of (a) condition on mean amplitude and (b) handedness on condition difference mean amplitude. This approach is designed to take into account the problem of multiple comparisons and data dependency in the statistical testing procedure. Potential condition differences (i.e. differences in the mean amplitude between the coherent and incoherent conditions) were examined separately for left- and right-handers. Potential handedness differences were examined by comparing the coherent minus incoherent mean amplitude difference observed in right- versus left-handers. In all instances, separate tests were conducted for the early and late time windows. For all tests, the number of permutations was set at 1000. Clusters of significant electrodes were built on the basis of spatial adjacency and significance thresholds exceeding *p* < 0.01. All electrode values within a cluster were required to have the same sign^54^ (i.e. positive or negative). The observed cluster with the largest mass (i.e. sum of all values within the cluster) was compared against the critical cluster value at the 99^th^ percentile of the null distribution.

#### Correlations

Correlation analyses were conducted to investigate the potential relationship between observed ERP activity and behavioural measures of vection in the coherent condition. After the ERP analyses identified the largest cluster of electrodes showing a significant mean amplitude difference between the coherent and incoherent conditions, the coherent condition mean amplitude values in the cluster electrodes were extracted. These were averaged together to give the cluster mean amplitude for the coherent condition. To enhance statistical power, the data for left- and right-handers were pooled. Spearman’s rho correlations, with Bonferroni-corrections for multiple comparisons, were conducted to quantify the relationship between the cluster mean amplitude and each behavioural measure of vection. Separate analyses were conducted for early and late windows.

## Results

### Behavioural measures of vection

A summary of the behavioural data is presented in Table 1. Overall, stronger vection was experienced in the coherent condition, compared to the incoherent condition, for both left- and right-handers. Specifically, more vection present trials, shorter onset latencies, longer vection durations, and higher strength scores were reported in the coherent condition. The behavioural measures of vection each showed relatively large variability, as is indicated by the interquartile ranges (see Table 1). All participants reported experiencing vection in the coherent condition. As vection was rarely present in the incoherent condition (median number of trials: 4.5 for left-handers, 4 for right-handers), measures of onset latency, duration, and strength were derived from very few trials. Consequently, statistical analyses comparing behavioural measures in the coherent versus incoherent condition were not conducted. Statistical contrasts comparing left- and right-handers found no significant differences in any behavioural measure of vection, in either the coherent or the incoherent condition.

**Table 1:** Behavioural measures of vection.

	Left-handers	Right-handers	Statistics		
			*Z*	*p*	*r*
**Vection presence (max = 50)**					
Coherent	48 (6)	47 (6.5)	1.02	0.31	0.13
Incoherent	4.5 (15)	4 (12)	− 0.11	0.92	− 0.01
**Onset latency (s)**					
Coherent	6.30 (4.61)	5.56 (4.62)	− 0.26	0.80	0.03
Incoherent	11.29 (9.43)	12.66 (8.51)	− 0.85	0.39	− 0.11
**Duration (s)**					
Coherent	12.62 (6.53)	14.29 (6.26)	− 0.41	0.68	− 0.05
Incoherent	4.56 (5.74)	3.86 (7.02)	0.16	0.87	0.02
**Strength (0–10)**					
Coherent	5 (3)	5.5 (4)	− 1.39	0.16	− 0.17
Incoherent	0 (0)	0 (0)	− 0.09	0.93	− 0.01

Presents median values (interquartile range) and statistical results from left- versus right-hander comparisons, in coherent and incoherent conditions. No significant differences were observed between the two groups. All participants reported vection perception in the coherent condition, whereas only a few subjects reported vection perception in the incoherent condition. Further, the vection experienced in the incoherent condition had longer onset latencies, shorter durations, and weaker subjective strength reports, when compared to the vection experienced in the coherent condition. *Z* = z-statistic, *p* = p-statistic, and *r* = effect size.

### Event related potentials (ERPs)

**Figure 1:**
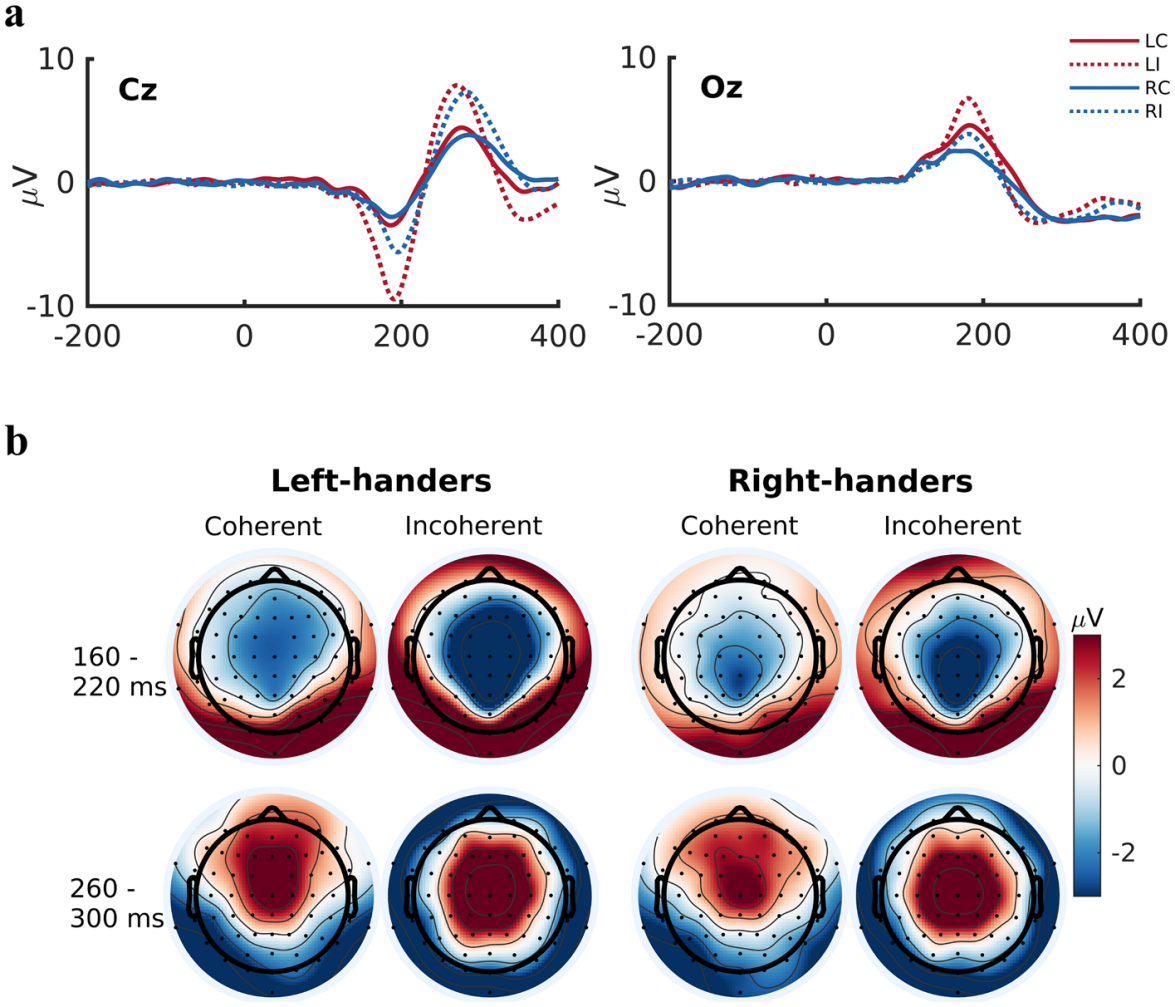
Overview of grand average ERP data. (**a**)  The grand average ERP waveforms in example electrodes Cz and Oz. Two clear peaks were observed across almost all electrodes following motion onset: an early peak around 160–220 ms and a later peak around 260–300 ms. LC = left-handers coherent, LI = left-handers incoherent, RC = right-handers coherent, RI = right-handers incoherent. (**b**)  Presents a topography of the mean amplitude values that were obtained in the early and late windows in the coherent and incoherent conditions, for left- and right-handers respectively. In both conditions, the early window was characterised by a pattern of fronto-central negativity (blue) and parieto-occipital positivity (red). This pattern reversed in the later window, with frontal positivity and parieto-occipital negativity in the coherent condition and a more central positivity with surrounding negativity in the incoherent condition. In general, larger amplitudes were observed in the incoherent condition and amongst the left-hander group.

Following motion onset, two clear ERP peaks were observable in coherent and incoherent conditions, in both left- and right-handers (see Fig. 1a, for example grand average ERPs in electrodes Cz and Oz). A general pattern of fronto-central negativity and parieto-occipital positivity was observed during the first peak, around 160–220 ms after motion onset (Fig. 1b). During the subsequent peak, around 260–300 ms, this pattern reversed in the coherent condition, with fronto-central positivity and parieto-occipital negativity being observed. The same peak in the incoherent condition was characterised by central positivity and surrounding negativity (Fig. 1b). In general, larger amplitudes were observed in the incoherent condition, in both left- and right-handers. As a group, left-handers exhibited larger amplitudes than right-handers.

### Coherent versus incoherent

**Figure 2:**
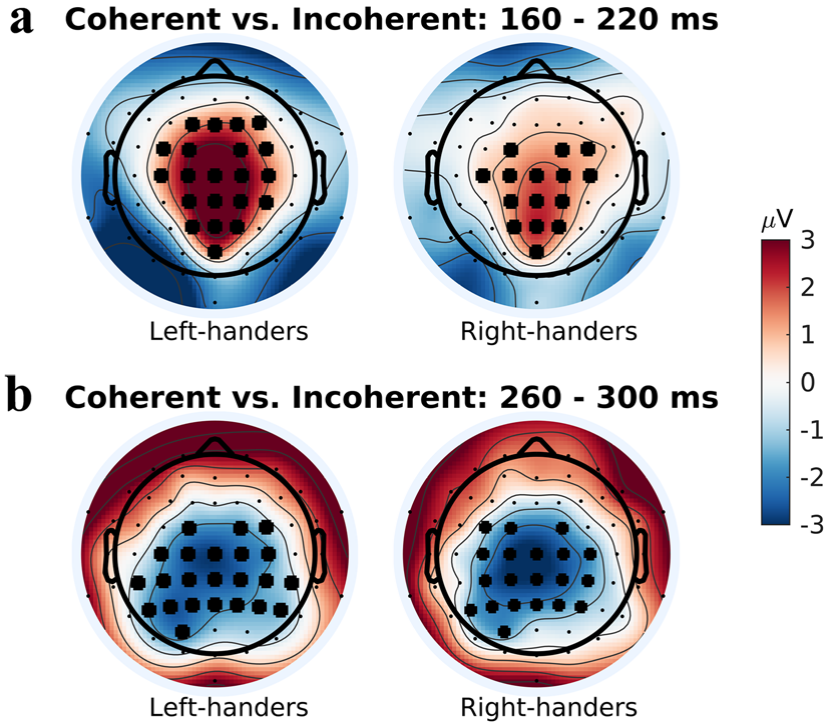
Mean amplitude in the coherent versus the incoherent condition. Cluster-based permutation tests were conducted to identify clusters of electrodes showing a significant difference in mean amplitude between the coherent and incoherent conditions in the early (160–220 ms) and late (260–300 ms) windows, for left- and right-handers respectively. The largest cluster of electrodes showing significant mean amplitude differences between the two conditions are indicated by large black dots over the respective electrode positions. For illustration purposes, condition differences are plotted as coherent mean amplitude minus incoherent mean amplitude, resulting in a net positive mean amplitude difference in the early window (red) and a net negative mean amplitude difference in the late window (blue). (**a**) Depicts the results in the early window for left- and right-handers. In both groups, a central cluster of electrodes, extending from parietal to frontal regions, exhibited significant condition differences, with attenuated mean amplitudes observed in the coherent relative to the incoherent condition. (**b**) Depicts the results in the late window for left- and right-handers. In both groups, a central cluster of electrodes, extending into centro-parietal regions, exhibited significant condition differences, with attenuated mean amplitudes observed in the coherent versus the incoherent condition.

Mean amplitude differences in the coherent versus the incoherent condition were assessed using cluster-based permutation tests in the early (160–220 ms) and late windows (260–300 ms), for left- and right-handers respectively (Fig. 2). In each case, significant differences were observed in multiple electrode clusters. The largest cluster (defined by the summed mass) exceeding a threshold of *p* < 0.01 is reported.

#### Left-handers, early window

**Figure 3:**
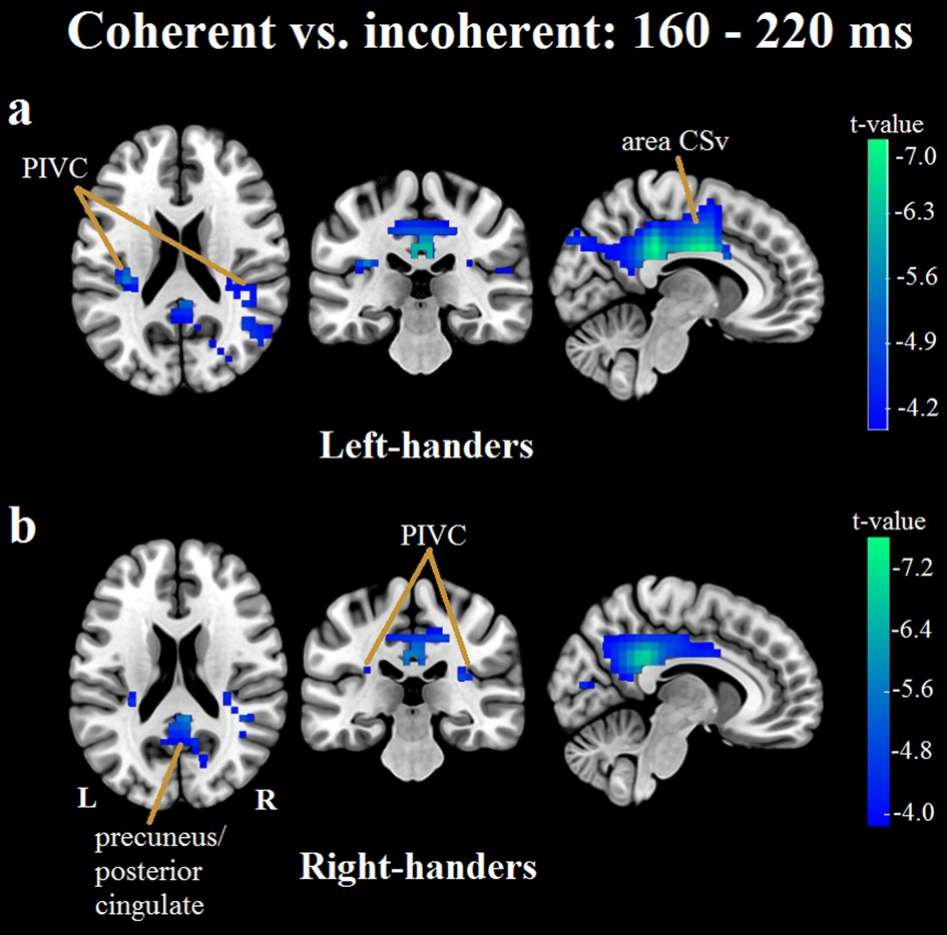
Visualisation of results from source localization analyses contrasting the estimated generators in the coherent versus the incoherent condition in the early (160–220 ms) window. In both left- and right-handers, estimated source activity differed across a large network consisting of the cingulate (including the cingulate sulcus visual (CSv) area), retrosplenial cortex, precuneus, sensorimotor, visual, parieto-occipital, frontal, and posterior insular/PIVC regions. Estimated source activity modulations in the cingulate, precuneus, and posterior insular/PIVC regions are depicted for left-handers (**a**) and right-handers (**b**). While left-handers exhibited bilateral activity in posterior insular/PIVC regions, right-handers showed a modest hemispheric asymmetry with more activity in the right posterior insular/PIVC regions (see Supplementary Fig. S2 for detailed visualization).

The largest cluster of electrodes exhibiting a significant condition difference was centrally located, centring around electrodes Cz/CPz and extending into both frontal and parietal electrodes (Fig. 2a). The cluster comprised electrodes showing a decreased mean amplitude in the coherent relative to the incoherent condition (Fig. 1b). Source localization analysis identified condition differences in estimated source activity across several brain regions. These regions include bilateral cingulate gyrus (mid/anterior and posterior divisions, and the cingulate sulcus visual (CSv) area); bilateral retrosplenial cortex; bilateral precuneus; sensorimotor regions (supplementary motor area, superior frontal gyrus, and pre- and post-central gyri); visual regions (bilateral cuneus, lateral occipital cortex, and occipital pole, and right intracalcarine cortex/V1); parieto-occipital regions (bilateral parietal lobule, right angular gyrus and supramarginal gyrus); frontal regions (right middle frontal gyrus and left inferior frontal gyrus); right middle temporal gyrus; and bilateral posterior insular/PIVC regions (left: parietal operculum including OP1/OP2, planum temporale, insular cortex; right: parietal operculum and planum temporale) (Fig. 3a). The maximum difference was found in the mid/anterior division of the cingulate gyrus.

#### Right-handers, early window

Similar to the left-handers, the largest cluster of electrodes showing a significant condition effect was centrally-located, stretching from parietal to centro-frontal electrodes (Fig. 2a). The cluster contained fewer electrodes and extended slightly less frontally when compared to the cluster observed in the left-hander group. Again, the cluster comprised electrodes showing a smaller mean amplitude in the coherent condition (Fig. 1b). Source localization analysis revealed condition differences in estimated source activity in several brain regions, including: bilateral cingulate gyrus (mid/anterior and posterior divisions, and area CSv); bilateral retrosplenial cortex; bilateral precuneus; bilateral sensorimotor precentral gyrus; visual regions (bilateral intracalarine cortex/V1, lateral occipital cortex, and right cuneus); parieto-occipital regions (bilateral parietal lobule, angular gyrus, and right supramarginal gyrus); and posterior insular/PIVC regions (left: planum temporale, insular cortex; right: planum temporale, insular cortex, parietal operculum, Heschel’s gyrus/OP2) (Fig. 3b). The maximum difference was observed in the posterior division of the cingulate gyrus.

#### Left-handers, late window

**Figure 4:**
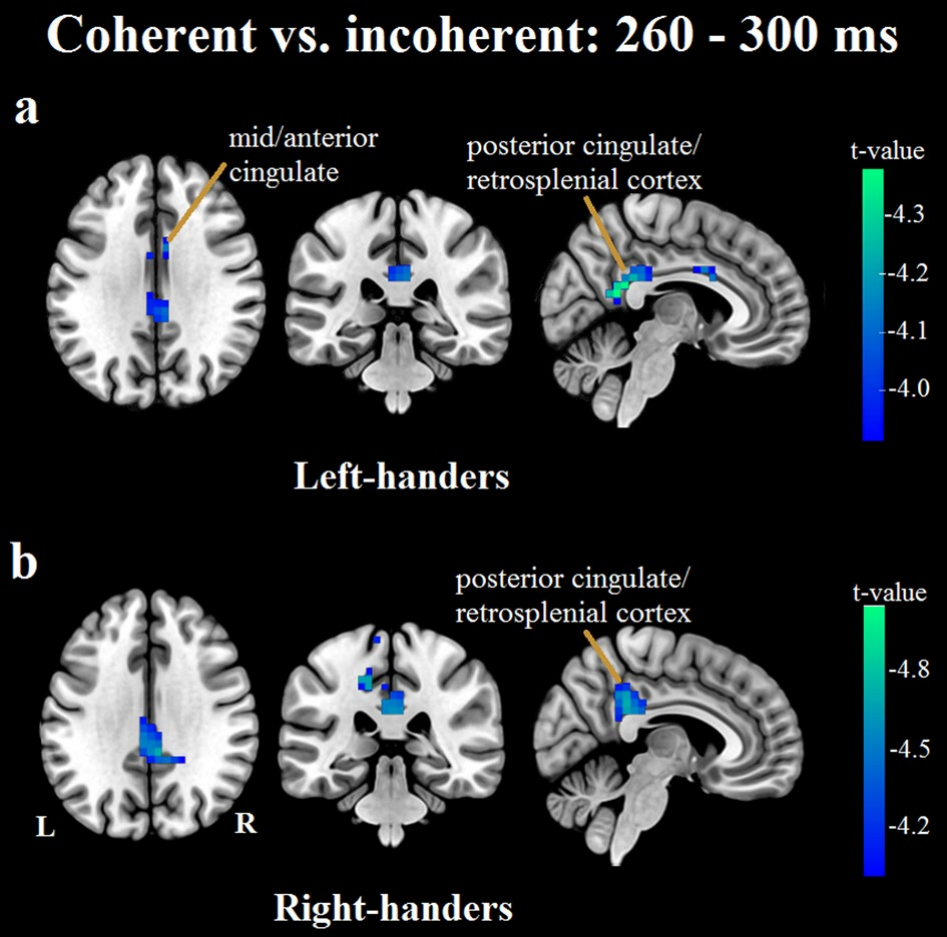
Visualisation of results from source localization analyses contrasting the estimated generators in the coherent versus the incoherent condition in the late (260–300 ms) window. In left-handers (**a**), estimated source activity modulations were observed in the posterior cingulate/retrosplenial cortex/precuneus, and the mid/anterior cingulate. In right-handers, estimated source activity modulations were identified in the posterior cingulate/retrosplenial cortex/precuneus, superior parietal lobule, pre- and post-central gyri, and (left) parieto-operculum/insular cortex. (**b**) Depicts the estimated source activity differences in the posterior cingulate/retrosplenial cortex/precuneus observed in right-handers.

The largest cluster of electrodes showing a significant condition difference was again centrally located, this time extending less frontally and more into parietal electrodes (Fig. 2b). Once more, the cluster comprised electrodes showing a smaller mean amplitude in the coherent, relative to the incoherent, condition. Source localization analysis identified significant condition differences in estimated source activity in bilateral posterior cingulate gyrus/retrosplenial cortex/precuneus and in bilateral mid/anterior cingulate gyrus (Fig. 4a). The maximum difference was located in the posterior cingulate gyrus/retrosplenial cortex.

#### Right-handers, late window

Again similar to the left-handers, the largest cluster showing condition differences was centrally located, with effects spreading into fronto-central and parietal electrodes (Fig. 2b). A smaller mean amplitude was observed across the cluster in the coherent condition. The source localization analysis revealed significant condition differences in estimated source activity in bilateral posterior cingulate gyrus/retrosplenial cortex/precuneus, right superior parietal lobule, left precentral gyrus, left postcentral gyrus, and left parieto-operculum/insular cortex (Fig. 4b). The maximum difference was located in the precuneus.

### Right-handers versus left-handers

Differences between left- and right-handers were examined by comparing the coherent minus incoherent mean amplitude difference between the two groups. Again, significant differences were assessed by means of cluster-based permutation tests in the early and late windows and the largest clusters exceeding a threshold of *p* < 0.01 are reported.

#### Early window

**Figure 5:**
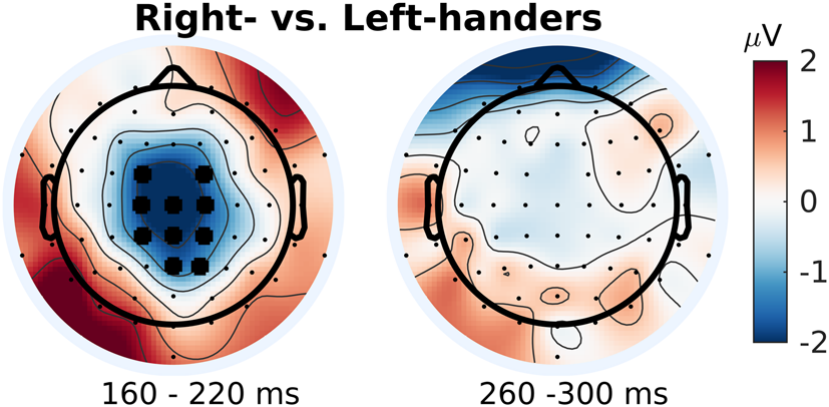
Condition difference (i.e. coherent mean amplitude minus incoherent mean amplitude) in right- versus left-handers. Cluster-based permutation tests were conducted to identify clusters of electrodes showing a significant condition difference between left- and right-handers in the early (160–220 ms) and late (260–300 ms) windows. For illustration purposes, condition differences between the two groups are plotted as right-hander condition difference minus left-hander condition difference. In the early window, a central cluster of electrodes (indicated by large black dots over respective electrode positions) exhibited a significant condition difference between the two groups, reflecting a larger mean amplitude difference in left- versus right-handers. That is, although both groups exhibited smaller mean amplitudes in the coherent relative to the incoherent condition, the difference between conditions was larger for left-handers. No significant differences were observed in the late window.

**Figure 6:**
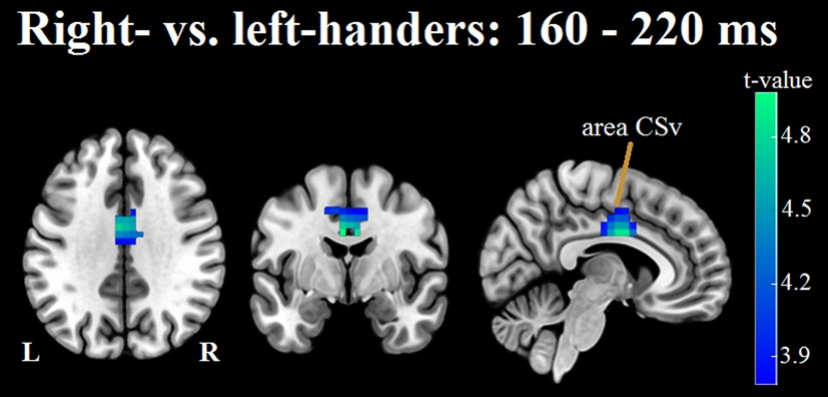
Visualisation of results from source localization analysis comparing the differences in estimated source activity between conditions in right- versus left-handers, during the early (160–220 ms) window. Results revealed that the estimated source activity exhibited in the coherent minus the incoherent condition differed between left- and right-handers primarily in area CSv.

A cluster of electrodes showing significant differences between left- and right-handers was centrally located, extending partially into fronto-central and centro-parietal electrodes (Fig. 5). This cluster reflects a larger mean amplitude difference in left-handers as compared to right-handers. More specifically, while both left- and right-handers showed a smaller amplitude in the coherent compared to the incoherent condition, this difference was greater for left-handers. Source localization analysis found significant differences in estimated source activity bilaterally in area CSv, extending along the mid/anterior cingulate and into the supplementary motor cortex (Fig. 6).

#### Late window

No significant differences were observed between the two groups.

### Behaviour-EEG correlations

The potential relationship between cluster mean amplitude and each behavioural measure of vection in the coherent condition was examined using Spearman’s rho correlations, with separate tests for early and late windows.

#### Early window

No significant correlations were observed between cluster mean amplitude and vection presence (ρ(55) = 0.11, *p* = 0.42), onset latency (ρ(55) = − 0.04, *p* = 0.75), or duration (ρ(55) = 0.09, *p* = 0.51). A moderate correlation was observed between mean amplitude and vection strength (ρ(55) = − 0.31, *p* = 0.018), however, this failed to reach statistical significance after Bonferroni correction for multiple comparisons was applied.

#### Late window

No significant correlations were observed between cluster mean amplitude and vection presence (ρ(55) = − 0.05, *p* = 0.71), onset latency (ρ(55) = 0.05, *p* = 0.72), vection duration (ρ(55) = 0.01, *p* = 0.92), or vection strength (ρ(55) = − 0.06, *p* = 0.65).

## Discussion

The present study used ERPs and source localization (eLORETA) to investigate the early neural processing of coherent vection-consistent visual motion stimulation against that of incoherent vection-inconsistent motion stimulation in left- versus right-handers. The behavioural results showed that prolonged exposure to the coherent stimulation resulted in moderately strong and reliable vection perception across participants, whereas prolonged exposure to the incoherent stimulation produced only infrequent reports of a relatively weaker vection. Onset of both coherent and incoherent motion stimulation generated ERP responses, with clear early (160–220 ms) and late (260–300 ms) peaks. In both windows, ERP analyses revealed reduced mean amplitudes in the coherent, relative to the incoherent, condition over predominantly central electrodes for both left- and right-handers (Fig. 2). In the early window, both left- and right-handers exhibited estimated source activity condition differences across a wide cortical network, including the cingulate and area CSv, the retrosplenial cortex, the posterior insula/PIVC, the precuneus, and visual, frontal and somatosensory regions (Fig. 3). In the late window, similar analyses identified estimated source activity differences primarily in the posterior cingulate/retrosplenial cortex/precuneus (Fig. 4). Comparison of left- and right-hander ERP activity in the early window found a greater mean amplitude difference between conditions amongst the left-hander group. Accompanying source localization analysis revealed that condition modulations of estimated source activity differed between left- and right-handers primarily in visual area CSv (Fig. 6). Despite these EEG differences, left- and right-handers reported comparable vection perception during prolonged motion stimulation exposure. Importantly, although vection was present and behaviourally measured in the current study, ERP and source localization findings are time-locked to motion onset, which occurs several seconds prior to vection onset. Thus, the reported ERP and source localization findings reflect the neural processes that occur *before* vection perception.

### The present findings in the context of previous ERP studies of vection

Prior ERP studies investigating *pre-vection* neural processes have emphasised two key findings: firstly, that motion onset of various movement patterns generates modulations of parieto-occipital P1/P2 and N2 components and secondly, that a tentative relationship exists between parieto-occipital N2 component amplitude and subjectively perceived vection strength/intensity^23–25^. Although parieto-occipital ERPs were observable in the present study (see Fig. 1b for the ERPs obtained at electrode Oz), our analyses indicated that a central cluster of electrodes exhibited the largest mean amplitude condition differences. The fact that our ERP effects were observed over predominantly central, rather than parieto-occipital, electrodes might be partially explained by our choice of control (i.e. incoherent) stimulation. Specifically, our control stimulation was designed to match the physical stimulus properties of the coherent stimulation as closely as possible and, although the control stimulation contained additional local sinusoidal motion, both stimulation types presented the same mean global velocity. In this way, we attenuated (but did not eliminate) ERP effects resulting from physical stimulus differences. This contrasts with the approaches taken in previous ERP studies that compared different centre-surround motion patterns^23,24^ or coherent versus random motion^25^ and found effects over parieto-occipital electrodes. Moreover, unlike previous ERP studies^23–25^, we employed large-field motion stimulation that covered a large field-of-view, resulting in more salient motion and possibly more robust, extensive recruitment of optic flow processing networks.

While previous ERP studies have reported a correlation between N2 component amplitude and subjective vection strength/intensity^23–25^, we observed only a moderate (ρ = − 0.31) non-significant correlation in the coherent condition between mean cluster amplitude in the early (160–220 ms) window and reports of subjective vection strength. It is worth noting here that correlations have also been reported between event-related spectral perturbations (ERSPs) in the delta, alpha, and beta bands and subjective vection intensity^22^. Importantly, these correlations between ERSP activity and vection intensity were observed at various time points both during and after the motion stimulation period, with the earliest correlation (between beta event-related desynchronization and vection intensity) occurring around 700–900 ms after motion stimulation onset. Altogether, these ERP and ERSP studies provide evidence to support a link between EEG activity modulations and subjective vection strength/intensity. Further, as is speculated by Palmisano and colleagues^22^, correlations that occur at different time points during/after motion stimulation likely reflect different stages of vection processing and/or perception (as indexed by EEG modulations), which each relate to subjective vection strength/intensity experience. As the correlation in our study was observed during coherent stimulation, just 160–220 ms after motion onset, we suggest that it reflects general and/or coherent motion detection rather than a specific distinction of vection-consistent motion stimulation. However, further research is needed to substantiate this proposal.

### Vection-consistent versus vection-inconsistent visual motion stimulation

Comparison of the ERPs evoked by coherent versus incoherent stimulation revealed consistent, relatively attenuated mean amplitudes in response to the vection-consistent coherent motion during both early and late windows. Accompanying source localization analyses, contrasting the estimated source activity in both conditions, identified largely distinct modulation patterns in the early and late windows. In the early window, estimated source activity differences were widespread across the cortex, including visual, sensorimotor, and multisensory vestibular networks. For both left- and right-handers, the strongest and most extensive condition modulations were observed in the cingulate cortex, which is part of the multisensory cortical vestibular network^33,34^. The cingulate has previously be found to prefer naturalistic self-motion over object-motion^13^ and has also been linked to vection perception^4,5,9^. Moreover, the bilateral cingulate sulcus visual (CSv) area has consistently been shown to be active during visual motion stimulation, only if the stimulation is self-motion compatible^55–57^. Notably, the identified modulations in left- and right-handers are located predominantly in mid and posterior cingulate regions, extending ventrally towards the border of the corpus callosum. Intracortical electrical stimulation of epilepsy patients in these regions was previously found to evoke subjective vestibular, interoceptive, somatosensory, and visual sensations^58^.

During the early window, left- and right-handers also exhibited condition modulations of estimated source activity in the posterior insular/PIVC regions (see Supplementary Fig. S2 for detailed visualization), which are core regions in the multisensory cortical vestibular network^33,34^. In line with previous findings demonstrating a thalamo-cortical hemispheric lateralization of the multisensory vestibular network during vestibular stimulation^29–35^, right-handers here exhibited a modest hemispheric asymmetry, with greater estimated source activity modulations in the right posterior insular/PIVC regions during visual motion stimulation. In contrast, left-handers exhibited bilateral estimated source activity modulations, with no asymmetry towards left posterior insular/PIVC regions during visual motion stimulation. This absence of left hemispheric dominance may reflect a weaker, more variable handedness preference amongst the left-handers. Indeed, as a group the left-handers exhibited more variable handedness laterality quotients (i.e. handedness scores) than the right-hander group (see Methods section for details). For both groups, estimated source activity modulations in the posterior insular regions included the planum temporale and parietal operculum, which have both previously been shown to respond more strongly to coherent/egomotion-compatible versus incoherent/egomotion-incompatible visual stimulation^56,59^.

In the late window, estimated source activity differences between the coherent and incoherent conditions were predominantly located in the posterior cingulate/retrosplenial cortex/precuneus for both left- and right-handers. The retrosplenial cortex is involved various aspects of spatial navigation and memory^60–62^, and has been found to be involved in the computation of heading direction during optic flow^63^ and active navigation^64^. Moreover, it is thought to be critically involved in the translation of information between allocentric (world-centred) and egocentric (self-centred) spatial reference frames^48,49,61,65–69^. Relatedly, the precuneus is thought to play a role in developing and maintaining concurrent egocentric and allocentric spatial reference frames^48,70^. There is also evidence to suggest that the posterior cingulate and retrosplenial cortex both contribute to our sense of self-location, with the posterior cingulate being particularly involved in the integration of neural representations of self-location and body ownership^71^. Altogether, our finding of condition modulations in these regions indicates heading computation differences between the two conditions, most likely due to stronger heading information in the vection-consistent coherent condition. Moreover, it suggests that vection-consistent coherent motion and vection-inconsistent incoherent motion interact differently with the network underlying egocentric and allocentric spatial reference frames.

### Left- versus right-handers

Our comparison of ERP condition differences in left- versus right-handers during the early window revealed a larger mean amplitude difference between coherent and incoherent stimulation in the left-handers. Accompanying source localization analysis in the same window identified group differences primarily in area CSv. It is now well established that area CSv not only prefers egomotion-compatible visual stimulation, but that it also shows suppressed or absent responses to egomotion-incompatible stimulation^13,55,56,72,73^. This raises the question of whether the larger ERP condition difference exhibited by the left-handers, partly reflects a greater suppression of CSv activity in response to incoherent versus coherent motion, as compared to that observed in the right-handers. Importantly, left- and right-handers reported comparable vection perception within both conditions, suggesting that the observed group differences in EEG activity are not due to one group experiencing more/less egomotion-compatibility in the coherent and/or incoherent condition, as compared to the other group.

In addition to visual egomotion stimulation, area CSv is also strongly responsive to vestibular stimulation^74,75^, making it a candidate location for the integration of visual and vestibular information related to self-motion^57,74^. Further, not only does area CSv receive afferent input from the vestibular system^74^, but functional and diffusion MRI indicate connectivity between area CSv and both ipsi- and contra-lateral posterior insular cortex (PIC)^76^. In recent years, evidence has emerged to suggest that the PIVC responds to vestibular input and is suppressed by visual motion stimulation whereas the posteriorly adjacent PIC comprises a distinct multisensory region responsive to both visual and vestibular inputs^35,77^. Due to the limited spatial resolution of EEG source localization methods, the present study does not attempt to distinguish between these two areas and rather refers to condition differences in the general posterior insular/PIVC region. Notably, the observed condition differences in this posterior insular/PIVC region are bilateral in left-handers and asymmetrical towards the right-hemisphere in right-handers, and occur in the same time window as the observed group differences in area CSv. Based on the outlined connectivity between these regions, a question arises about whether (and how) the handedness-dependent condition differences in the posterior insular/PIVC region might relate to the group differences in area CSv. We speculate that different handedness-dependent activity patterns in the posterior insula during coherent versus incoherent visual stimulation might result in differential afferent (and/or feedback) signals with area CSv, thus resulting in the observed EEG group differences. Importantly, given the comparable perceptual reports from left- and right-handers such connectivity differences would appear to be behaviourally insignificant, at least in the context of the present study. However, future work is required to validate this proposed relationship between handedness and activity in the posterior insula and CSv areas, and to clarify whether such a relationship has any implications for vection perception.

### Limitations and future directions

A possible limitation of the present study is that it does not address the potential contribution of torsional eye movements to the observed effects. Although participants maintained central fixation, both conditions included continuous roll motion capable of triggering mild torsion. As we did not measure torsion, we cannot exclude the possibility that the global motion in the incoherent condition resulted in weaker, less frequent torsion relative to that in the coherent condition. Although we did not observe condition differences in oculomotor-specific regions, area CSv is also thought to be involved in the integration of oculomotor and visual motion signals related to self-motion^47,78^. As such, it is possible that torsional eye movements contribute in small part to our condition difference findings.

Further, it should also be acknowledged that distributed source localization of EEG data attempts to solve an ill-posed problem using specific assumptions. Although the source localization results presented here are in line with previous knowledge, it should be kept in mind that these localization results lack the spatial certainty provided by other imaging methods.

Lastly, the extent to which the findings of the present study will generalize to visual motion stimulation and/or vection in other planes, axes, and contexts remains a question for future research. For example, during actual roll motion optic flow is accompanied by continuous vestibular information, whereas constant velocity forward motion generates continuous optic flow but limited vestibular information (i.e. primarily at acceleration). Similarly, different types of visual motion stimulation and/or vection are likely to involve differing visual-vestibular interactions and neural processes. In line with this, some evidence indicates that that the same visual motion stimulus can elicit differences in vection perception^79^ and vection-related EEG activity^20^ when an individual is in an upright versus a supine position (i.e. thereby changing the vestibular information with respect to the gravity vector).

## Conclusions

We observed that vection-consistent motion stimulation evoked consistently attenuated central ERPs, relative to incoherent control stimulation, for both left- and right-handers. Early ERP differences were accompanied by estimated source activity modulations across a large cortical network comprising visual, sensorimotor, and multisensory vestibular regions, whereas modulations accompanying later ERP differences were limited to the posterior cingulate/retrosplenial cortex/precuneus. In contrast to right-handers, left-handers exhibited a larger ERP condition difference. This was accompanied by group differences in the cingulate sulcus visual (CSv) area, suggesting that handedness may influence both ERP and area CSv responses to vection-consistent and vection-inconsistent visual motion stimulation.

## Supplementary Information

Supplementary Information.

## Data Availability

Example data and code is available from the corresponding author on reasonable request.
