# Supplementary material for: EEG analysis of the visual motion activated vection network in left- and right-handers
Source: Sci Rep. 2022 Nov 15;12:19566. doi: 10.1038/s41598-022-21824-x (PMC9666650; doi:10.1038/s41598-022-21824-x)
Supplement: Supplementary file 1 — Supplementary Information. [file 41598_2022_21824_MOESM1_ESM.pdf]

EEG analysis of the visual motion activated vection network in left- and right-handers

Michaela McAssey*[1-4], Thomas Brandt, MD[2-4], Marianne Dieterich, MD[1-5]

[1] Department of Neurology, University Hospital, Ludwig-Maximilians-Universität, München, Germany

[2] German Center for Vertigo and Balance Disorders-IFB, University Hospital, Ludwig-Maximilians-Universität, München, Germany

[3] Graduate School of Systemic Neurosciences (GSN), Ludwig-Maximilians-Universität, München, Germany

[4] RTG 2175, Perception in Context and its Neural Basis, Ludwig-Maximilians-Universität, München, Germany

[5] Munich Cluster for Systems Neurology (SyNergy), München, Germany

*Corresponding Author:

Michaela McAssey,

Department of Neurology, Ludwig-Maximilians-Universität, München

Fraunhoferstraße 20

82152 Planegg, Germany

Mail: michaela.mcassey@med.uni-muenchen.de

SUPPLEMENTARY INFORMATION

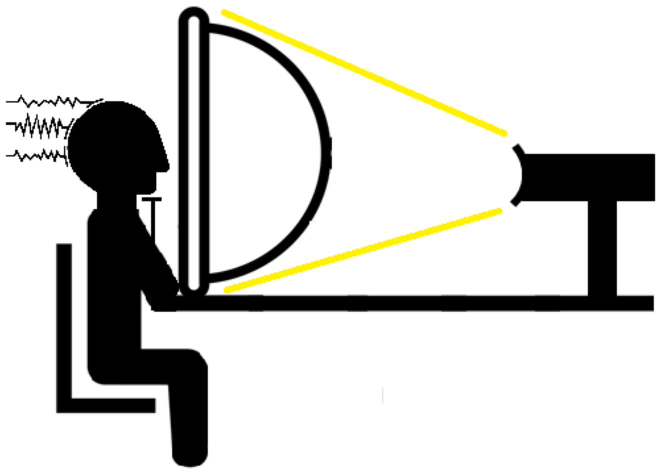

Figure S1

Schematic illustration of the experimental setup. Participants sat, with their chin on a chin rest in a dark room. The stimuli were projected onto a custom-built dome (diameter: 75 cm). The distance between the apex of the dome and the participant's nasion was 31 cm. The stimuli rotated around the line of sight, subtending a visual angle of 100°.

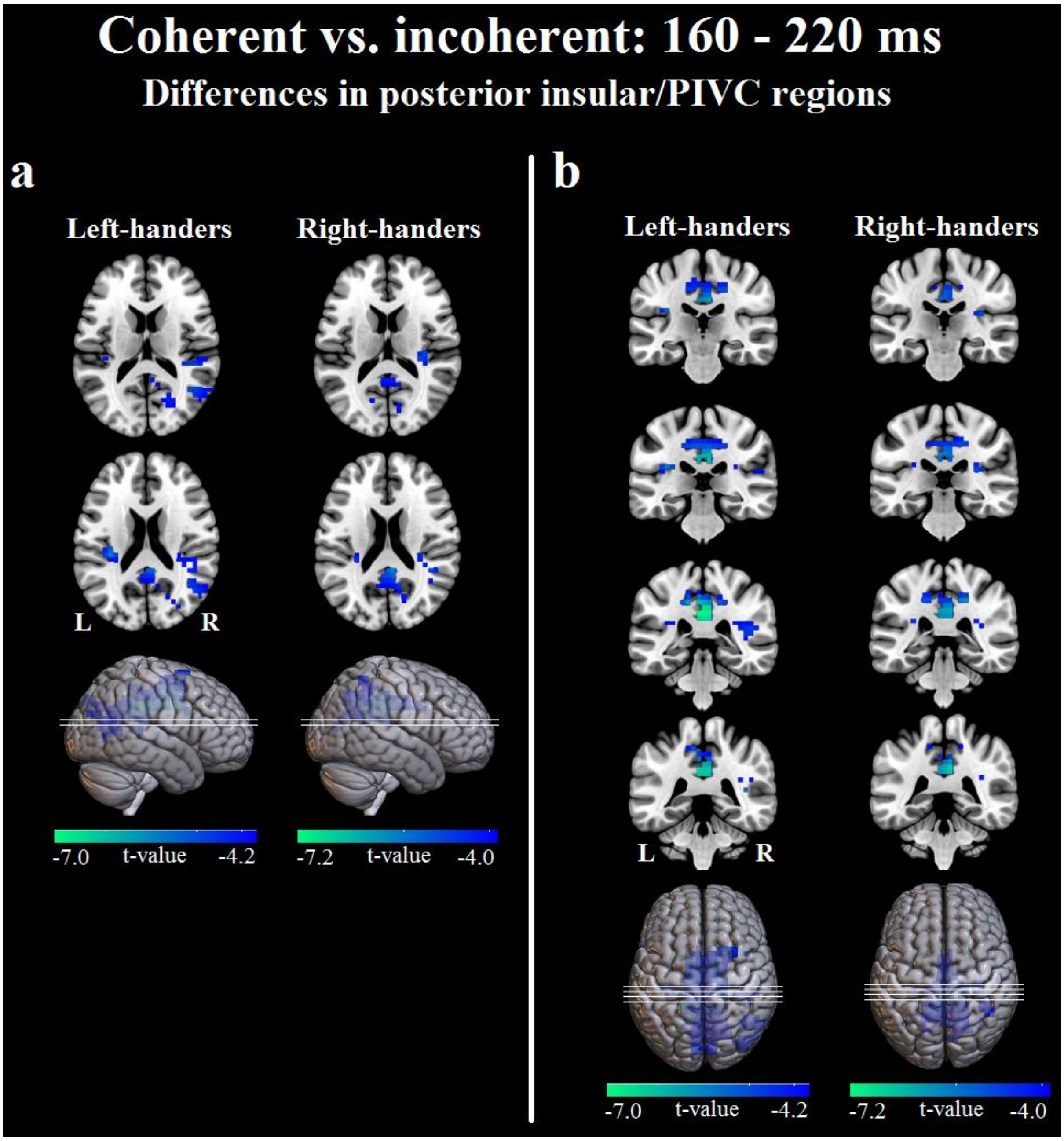

Figure S2

Visualisation of results from source localization analyses contrasting the estimated generators in the coherent versus the incoherent condition in the early (160 – 220 ms) window, focusing on the posterior insular/PIVC regions. Results are presented from an axial (Panel a) and a coronal (Panel b) view. Left-handers exhibited bilateral estimated source activity modulations in posterior insular/PIVC regions (left hemisphere: parietal operculum including OP1/OP2, planum temporale, insular cortex; right hemisphere: parietal operculum and planum temporale). In contrast, right-handers showed a modest hemispheric asymmetry towards right posterior insular/PIVC regions (left hemisphere: planum temporale and insular cortex; right hemisphere: planum temporale, insular cortex, parietal operculum, Heschel’s gyrus/OP2). To quantify hemispheric differences, the number of condition modulated

voxels in the right hemisphere posterior insular/PIVC region was divided by that in the left hemisphere, for left- and right-handers respectively. This resulted in a right-to-left hemisphere ratio of 1.63 amongst left-handers and a right-to-left hemisphere ratio of 3.5 amongst right-handers.
